# BMPR2 promotes invasion and metastasis via the RhoA-ROCK-LIMK2 pathway in human osteosarcoma cells

**DOI:** 10.18632/oncotarget.17382

**Published:** 2017-04-24

**Authors:** Shidong Wang, Tingting Ren, Guangjun Jiao, Yi Huang, Xing Bao, Fan Zhang, Kuisheng Liu, Bingxin Zheng, Kunkun Sun, Wei Guo

**Affiliations:** ^1^ Musculoskeletal Tumor Center, Peking University People's Hospital, Beijing, 100044, China; ^2^ Beijing Key Laboratory of Musculoskeletal Tumor, Beijing, 100044, China; ^3^ Department of Orthopedics, Qilu Hospital of Shandong University, Jinan, 250012, China; ^4^ Department of Pathology, Peking University People's Hospital, Beijing, 100044, China

**Keywords:** BMPR2, phosphorylation, LIMK2, metastasis, osteosarcoma

## Abstract

Bone morphogenetic protein receptor 2 (BMPR2) has been identified in several types of cancer. However, its role in osteosarcoma is largely unknown. We systematically investigated the role of BMPR2 in osteosarcoma cell lines, human tissue samples and xenograft models. The relationship between BMPR2 expression and osteosarcoma patients’ survival was investigated by bioinformatics and clinical data. Wound healing assay and transwell assay were used to detect the changes of cell migration and invasion ability after BMPR2 transfection. In addition, downstream phosphoproteins were analyzed by iTRAQ-based phosphoproteomic analysis and verified by western blotting. *In vivo*, the effects of BMPR2 on the growth, formation and metastasis of 143B cells were observed by orthotopic transplantation of nude mice. Here, we demonstrated that BMPR2 expression was elevated in a majority of osteosarcoma tissues compared with normal bone tissue. Osteosarcoma patients with greater BMPR2 expressing level showed a poor overall survival. The depletion of BMPR2 in 143B cells markedly reduced the invasive capacity *in vitro* and metastatic potential *in vivo*. Mechanistically, we found that LIM domain kinase 2 (LIMK2) was phosphorylated and activated by BMPR2 and that this event was crucial for activation of the BMPR2-mediated signal pathway in osteosarcoma cells. Additionally, we demonstrated that BMPR2 could active LIMK2 through the RhoA/ROCK pathway and could also interact with LIMK2 directly. Taken together, our study revealed that BMPR2 functions as a prometastatic oncogene *in vitro* and *in vivo* with the activation of the RhoA-ROCK-LIMK2 pathway and may represent a potential therapeutic target for osteosarcoma.

## INTRODUCTION

Osteosarcoma is the most common primary bone malignancy diagnosed in children and adolescents and has a high propensity for local invasion and distant metastasis [1–4]. Advances of neoadjuvant chemotherapy and the refinements of surgical techniques have greatly raised the 5-year survival rate of patients with localized osteosarcoma. However, in pulmonary metastases patients, little has changed and the survival is usually < 25%[2]. Thus, it is urgent to identify new potential targets or factors that govern metastasis and to develop novel therapeutic tactics for osteosarcoma management.

Bone morphogenetic proteins (BMPs) are multifunctional proteins that are members of the transforming growth factor β (TGF-β) superfamily [5, 6]. They play an important role during development and regulate many processes, including cellular proliferation, adhesion, migration, differentiation, inflammation, and apoptosis [7–9]. Mature BMP ligands bind to bone morphogenetic protein receptor 1 and 2 (BMPR1 and BMPR2) on the cell surface, leading to the phosphorylation of Smad1/5/8 and the interaction with Smad4. Then the Smad signaling pathway becomes activated [5, 10, 11]. BMP signaling can also follow a Smad-independent pathway through several intracellular mediators such as p38 MAPK, ERK and JNK [5].

BMP signaling plays a dual role during regulating tumor formation and metastasis. As an important component of BMP signal transduction, BMPR2 plays a pivotal role in tumor development. Accumulating research has revealed that aberrant expression of BMPR2 is implicated with many cancers [12–17]. In some tumors, BMPR2 plays a role as a tumor suppressor gene. For example, conditional inactivation of BMPR2 in colon stroma leads to more pronounced colon epithelial hyperplasia and the formation of polyps [13]. Loss of expression of BMPR2 and its poor prognosis have been demonstrated in human prostate cancer [12]. In addition, compared with normal tissue, lack of BMPR2 expression is more common in bladder transitional cell carcinoma tissues [18]. In some other circumstances, BMPR2 was illustrated as an oncogene. Pouliot F et al [19] demonstrated that the downregulation of BMPR2 obviously inhibited the proliferation and activity of breast cancer cells. And in chondrosarcoma cells, BMPR2 silencing could induce apoptosis and autophagy by a XIAP-mediated pathway [16]. Further, BMPR2 mRNA was increased in osteosarcoma and was correlated with metastasis in osteosarcoma [20]. However, there is little knowledge about the concrete mechanism of action of BMPR2 in osteosarcoma.

In this study, we investigated the relationship between BMPR2 and its clinical outcomes. The role of BMPR2 in human osteosarcoma cell migration, invasion and metastasis was examined *in vitro* and *in vivo*. The underlying mechanism of BMPR2-mediated cell invasion was also investigated.

## RESULTS

### BMPR2 expression is elevated in osteosarcomas and is associated with poorer outcome

**Figure 1:**
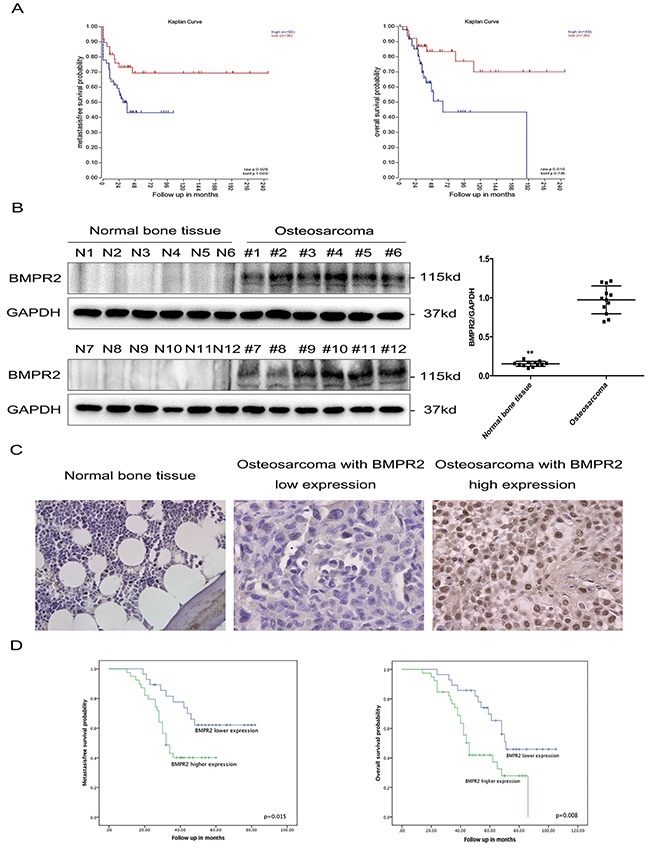
Elevated BMPR2 expression is predictive of poor outcome in osteosarcoma patients **(A)** (Left) Kaplan-Meier metastasis-free survival curve for osteosarcoma patients in the GSE33383 database showing the impact of BMPR2 expression on outcome. (Right) Kaplan-Meier curves of overall survival for osteosarcoma patients in the GSE33383 database based on BMPR2 expression. **(B)** Western blotting showing that BMPR2 is expressed in osteosarcoma but not in normal bone tissues. The data on BMPR2 expression levels in osteosarcoma and normal bone tissues are presented as the mean ± S.D. (n=12). **P<0.01. **(C)** IHC staining for BMPR2 expression in osteosarcoma samples and normal bone tissue. Representative images were photographed at ×400. **(D)** (Left) Kaplan-Meier curves showing the metastasis-free survival of 67 osteosarcoma patients. (Right) Kaplan-Meier curves showing the impact of BMPR2 expression on overall survival for 67 osteosarcoma patients.

To investigate the relationship between BMPR2 levels and the outcomes in osteosarcoma, we first searched for publically available datasets. 88 cases in the GSE33383 dataset were able to be involved in the survival analysis, which indicated high BMPR2 expression may be implicated with decreased metastasis-free survival and overall survival in osteosarcoma (Figure 1A).

We further detected BMPR2 protein expression in 12 osteosarcoma tissues and 12 normal bone tissues by western blotting, and found that compared to normal bone tissue, BMPR2 expression was significantly higher in osteosarcoma (Figure 1B).

Immunohistochemistry detection of BMPR2 was performed on 67 osteosarcoma samples, which was divided into a high and low expression group according to the cut-off value (median staining score: 4.0) (Figure 1C). Yellow or brown indicated positive expression of the marker. The location of BMPR2 expression was identified in the nucleus and cytoplasm. The clinical and pathological characteristics are presented in Table 1. High levels of BMPR2 were found to be associated with advanced Enneking stage (*p*=0.006) and lung metastasis (*p*=0.007) (Table 1). However, there were no obvious differences between BMPR2 expression and gender, age, tumor location, and histological types.

**Table 1:** Relationship between BMPR2 expression level and clinicopathological features in osteosarcoma (n = 67)

Variables	Cases	BMPR2 lower expression(n=28)		BMPR2 higher expression (n=39)		P-value
		N	%	N	%	
**Gender**						0.246
Male	36	15	53.6	21	53.8	
Female	31	13	46.4	18	46.2	
**Age at diagnosis**						0.725
≤20 years	39	17	60.7	22	56.4	
>20 years	28	11	39.3	17	43.6	
**Tumor location**						0.426
Femur	24	10	35.7	14	35.9	
Tibia	16	7	25.0	9	23.1	
Humerus	12	7	25.0	5	12.8	
Others	15	4	14.3	11	28.2	
**Histological types**						0.534
Osteoblastic	38	18	64.3	20	51.3	
Chondroblastic	16	6	21.4	10	25.6	
Others	13	4	14.3	9	23.1	
**Enneking stage**						**0.006**
I + II	45	24	85.7	21	53.8	
III	22	4	14.3	18	46.2	
**Lung metastasis**						**0.007**
Yes	37	10	35.7	27	69.2	
No	30	18	64.3	12	30.8	

The prognostic significance of BMPR2 expression was further investigated. As shown in Figure 1D, high expression of BMPR2 was found corresponding to shorter metastasis-free time (*p*=0.015) and overall survival time (*p*=0.008). Univariate and multivariate analyses were conducted to observe the factors related to the prognosis of osteosarcoma patients. Our data revealed that BMPR2 level, advanced Enneking stage and lung metastasis were correlated with the metastasis-free and overall survival of osteosarcoma patients (Table 2 & 3). These results indicated that osteosarcoma patients with high expression of BMPR2 were more likely to have a poor prognosis.

**Table 2:** Univariate and multivariate analysis of clinicopathological parameters associated with metastasis-free survival in osteosarcoma patients

Variables	Univariate analysis		Multivariate analysis	
	HR (95%CI)	*P* value	HR (95%CI)	*P* value
Gender (male vs. female)	1.040(0.524-2.065)	0.910		
Age (≤20 vs. >20 years)	0.624(0.302-1.287)	0.201		
Tumor location	0.849(0.627-1.150)	0.290		
Histological classification	0.647(0.389-1.078)	0.095		
Enneking stage (I +II vs III)	7.122(3.285-15.441)	<0.001	3.679(1.609-8.413)	0.002
Lung metastasis (yes vs. no)	13.285(4.534-38.932)	<0.001	10.019(3.263-30.765)	<0.001
BMPR2 (high vs. low)	2.493(1.161-5.353)	0.019	1.832(1.008-3.925)	0.041

**Table 3:** Univariate and multivariate analysis of clinicopathological parameters associated with overall survival in osteosarcoma patients

Variables	Univariate analysis		Multivariate analysis	
	HR (95%CI)	*P* value	HR (95%CI)	*P* value
Gender (male vs. female)	1.142(0.606-2.151)	0.682		
Age (≤20 vs. >20 years)	0.934(0.495-1.761)	0.833		
Tumor location	0.947(0.722-1.240)	0.691		
Histological classification	0.792(0.521-1.203)	0.274		
Enneking stage (I +II vs III)	9.005(4.218-19.225)	<0.001	5.949(2.605-13.586)	<0.001
Lung metastasis (yes vs. no)	5.962(2.771-12.831)	<0.001	4.824(2.085-11.161)	<0.001
BMPR2 (high vs. low)	2.419(1.227-4.771)	0.011	2.013(1.045-4.162)	0.037

### Effect of BMPR2 depletion or overexpression on the proliferation of osteosarcoma cells

**Figure 2:**
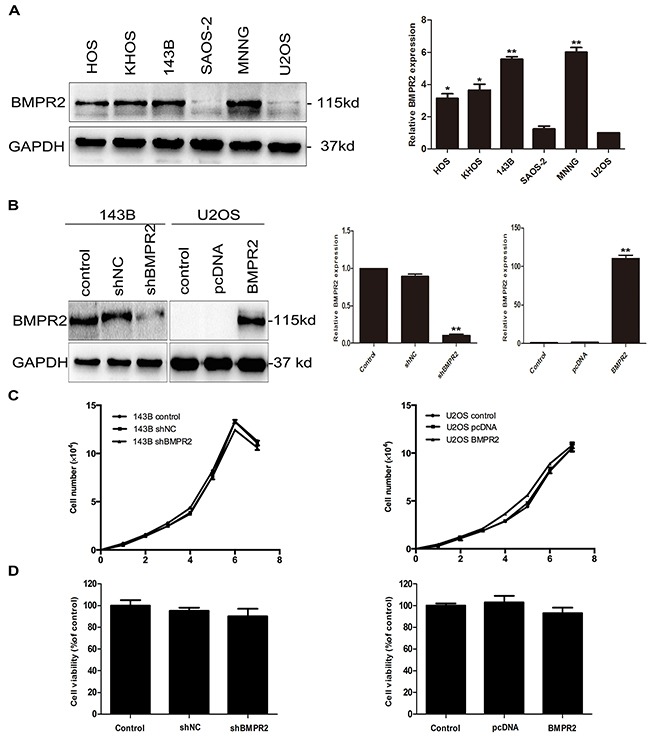
Effect of BMPR2 expression on the proliferation of osteosarcoma cells **(A)** Endogenous expression of BMPR2 in a panel of osteosarcoma cell lines was analyzed by western blotting and real-time PCR. **(B)** The transfection efficiency of BMPR2 in 143B cells and U2OS cells was evaluated by real-time PCR and western blotting. **(C)** Cell proliferation was detected after transfection in 143B and U2OS cells. **(D)** The osteosarcoma cell viabilities after BMPR2 transfection were assayed by MTS. Data are presented as the mean ± S.D. (n=3). *P<0.05 and **P<0.01.

Endogenous BMPR2 mRNA and protein levels were examined in six osteosarcoma cell lines, among which HOS, KHOS, 143B, MNNG cell lines showed significantly higher levels of BMPR2 mRNA and protein than U2OS cells (Figure 2A). However, there was no difference in BMPR2 expression in terms of mRNA and protein levels between SAOS-2 and U2OS cells. 143B and U2OS cell lines were chosen for further study.

To examine the function of BMPR2 in osteosarcoma cells, the shRNA and overexpression vector targeting BMPR2 were transfected into 143B and U2OS cells, respectively. Western blotting and RT-PCR were used to detect transfection efficiency. BMPR2-shRNA was able to significantly suppress BMPR2 expression in 143B cells. Further, exogenous expression of BMPR2 was efficiently upregulated in U2OS cells after adenoviral transient transfection (Figure 2B).

We next observed the proliferation of cells after transfection with shRNA or overexpression vector. As is shown in Figure 2C, there were no changes in cell growth rates in both types of cells. We have also observed cell viabilities through the MTS assay and found that the cell viabilities of osteosarcoma cells were no significant difference whether in 143B cells or U2OS cells (Figure 2D, *p*=0.329 and *p*=0.712, respectively). This indicated that the alteration of BMPR2 expression was not correlated with the proliferation of 143B or U2OS cells.

### Suppression of BMPR2 decreases the migration and invasive capacity of osteosarcoma cells

**Figure 3:**
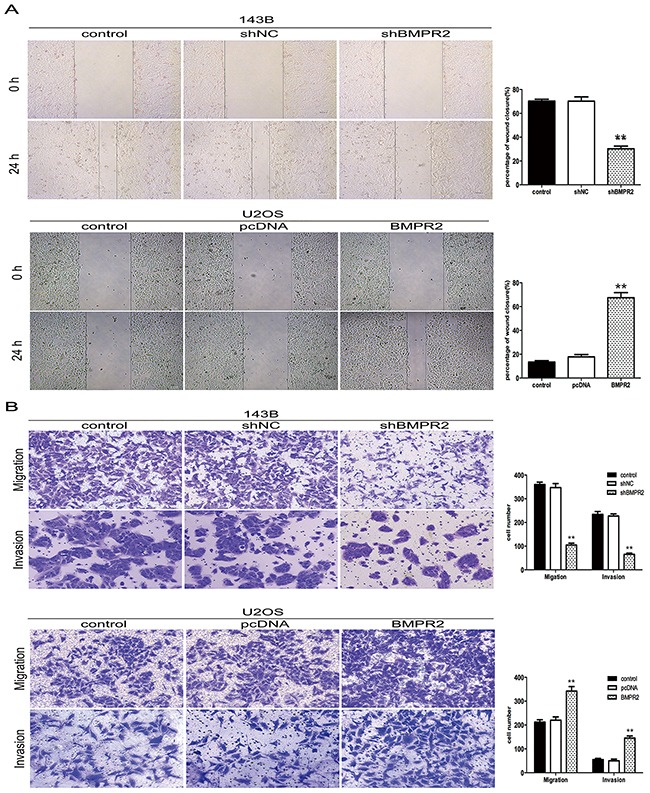
BMPR2 silencing decreases osteosarcoma cell migration and invasion **(A)** Wound-healing assay for 143B and U2OS cells after transfection for 24 h. **(B)** Transwell migration assay and Matrigel invasion assay for 143B and U2OS cells after transfection for 24 h. Cells were stained with crystal violet (magnification: ×200). Data are based on at least three independent experiments, and shown as the mean ± S.D. (**P < 0.01 compared with control).

As no influence of BMPR2 levels on osteo-sarcoma cells’ proliferation, it was interesting to examine whether BMPR2 affected the migration and invasion of 143B and U2OS cells. The wound healing assay showed that the cells in the shBMPR2 group had less motility than the corresponding control cells (Figure 3A). In contrast, U2OS cells’ motile abilities were enhanced by BMPR2 overexpression (Figure 3A). Additionally, the results of the transwell assay showed that the migrated and invaded cells were more likely concentrated on the BMPR2-overexpression group in U2OS cells (Figure 3B). A much lower number of 143B cells were found in the BMPR2-silenced group than that in control group (Figure 3B). The changes in cell motility and invasion suggest a pro-metastasis role for BMPR2 in osteosarcoma cells.

### BMPR2 silencing increases the mesenchymal-epithelial transition (MET) in osteosarcoma cells

**Figure 4:**
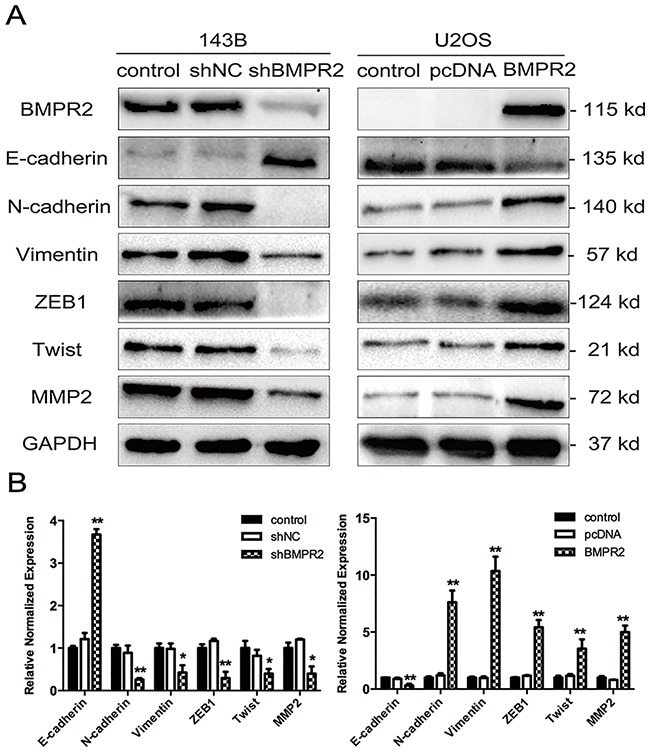
Effect of BMPR2 expression on MET progression **(A)** MET markers as well as MMP2 were assayed using western blotting in 143B and U2OS cells after transfection. **(B)** In parallel, real-time PCR was used to detect the mRNA levels of MET markers and MMP2. The results of three independent experiments are presented as the means ± SD. (*P<0.05 and **P<0.01 compared with control group).

A change in morphology from mesenchymal-like to epithelial-like was observed in 143B cells after BMPR2 silencing (data not shown), so we investigated whether other characteristics of mesenchymal cells could be regulated by BMPR2 expression. Therefore, mRNA and protein expression of MET markers were investigated by the methods of real-time PCR and western blotting. The primers used for quantitative real-time PCR were presented in Supplementary Table 1. E-cadherin, as the most important epithelial marker, was higher in shBMPR2 cells compared with the control group (Figure 4A). Accompanied with BMPR2 silencing, N-cadherin and vimentin, the mesenchymal markers, were down-regulated in 143B cells (Figure 4A). Moreover, the expression of MMP2 and some MET-inducing transcription factors such as ZEB1 and Twist1 were also decreased significantly in the BMPR2-depletion group when compared to the control group (Figure 4A). We further observed the upregulation of MMP2 and mesenchymal proteins (N-cadherin, vimentin, Twist1, ZEB1) and the down regulation of epithelial marker (E-cadherin) in U2OS cells, which are accompanied with BMPR2 overexpression (Figure 4A). The results of the corresponding mRNA levels of MET markers were consistent with protein levels in both types of cells (Figure 4B). These data demonstrate that BMPR2 silencing increases MET progression in osteosarcoma cells.

### Identification of genes regulated by BMPR2 via quantitativephosphotyrosine proteomic analysis

**Figure 5:**
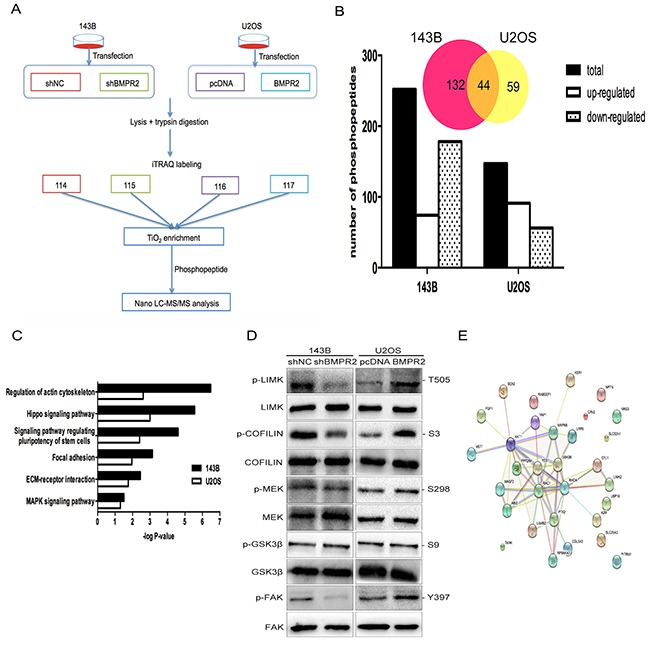
A quantitative phosphotyrosine proteomic analysis was performed as described in the materials and methods **(A)** Flow diagram of quantitative phosphotyrosine proteomic analysis. **(B)** The bar chart shows the numbers of phosphopeptides that are significantly regulated. Venn diagram presents the number of overlapped phosphoproteins according to the phosphopeptides. **(C)** The pathways revealed by GO enrichment analysis of BMPR2 regulated phosphoproteins were shown according to their *p* values in the experiment. **(D)** Validation of the results of phosphoproteomics using western blotting. **(E)** String analysis of differentially expression phosphoproteins that were identified.

To elucidate the underlying mechanism, we detected the dynamics of BMPR2-dependent protein phosphorylation in both 143B and U2OS cells through quantitative phosphoproteomics. The flow diagram of the method was presented in Figure 5A.

A total of 1458 phosphopeptides were detected in two independent biological replicates among four groups. Then, we analyzed the identified phosphopeptides. As a result, 252 phosphopeptides spanning 305 phosphorylation sites in 176 proteins were detected in 143B cells. Moreover, in U2OS cells, 147 phosphopeptides spanning 168 phosphorylation sites in 103 proteins were examined. Phosphopeptides numbers in both cell lines were displayed in Figure 5B. Only the overlapped protein between 143B and U2OS cells were used for assessment of functional changes (Figure 5B & Supplementary Table 2). The result for the protein-protein interaction network was displayed in Figure 5E.

To investigate the combined downstream pathway that was associated with BMPR2, we further conducted GO enrichment analysis. The overlapping enriched pathways in both 143B and U2OS cell lines were presented in Figure 5C. This result suggests that the BMPR2 gene was closely associated with actin cytoskeletal regulation and the focal adhesion pathway. Furthermore, the changes in phosphorylated proteins obtained from iTRAQ analysis were confirmed by western blotting. Consistent with our pathway analyses, the expression of p-LIMK2 and p-cofilin were decreased with BMPR2-depletion in 143B cells compared to the shNC group (Figure 5D, *p*<0.001 and *p*=0.013, respectively). Compared with the control group, BMPR2 overexpression in U2OS cells accompanied by increased p-LIMK2 and p-cofilin (Figure 5D, *p*=0.008 and *p*<0.001, respectively), but no changes were found in the corresponding total protein. This result further confirmed the reliability of phosphotyrosine proteomic analysis.

### BMPR2 activates ROCK/LIMK2 signaling through a Smad- independent pathway

**Figure 6:**
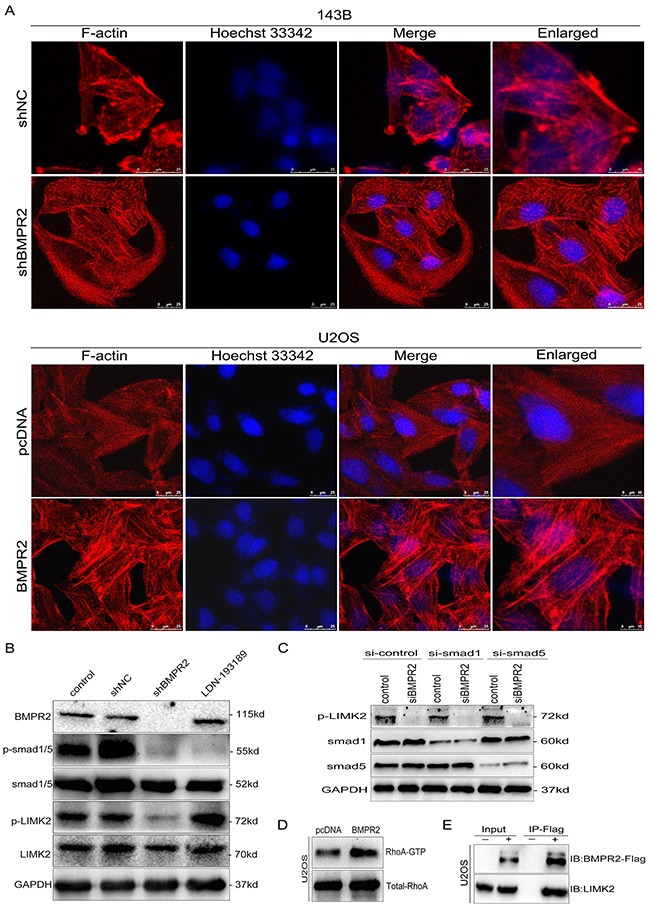
BMPR2 activates ROCK/LIMK signaling through a Smad-independent pathway **(A)** Cytoskeletal assay of 143B and U2OS cells was visualized by confocal microscopy. Representative images were shown. Cell nuclei were stained with Hoechst33342. Scale bar represents 25 μm or 10 μm. **(B)** BMPR2-silencing attenuates p-LIMK expression through the Smad-independent pathway. **(C)** Smad1 and Smad5 are not required for BMPR2-depleted down-regulation of p-LIMK2. **(D)** GTPase activation assay validated the changes of RhoA in U2OS cells. **(E)** Interaction of LIMK2 with BMPR2 in U2OS cells. U2OS cells were transfected with Flag-BMPR2 and Flag-control. Cell lysates were immunoprecipitated with Flag-beads, and immunoblotted with anti-BMPR2 (top panel) or anti-LIMK2 antibodies (bottom panel).

As the actin-binding protein, LIMK2 and cofilin are important regulators of actin reorganization [21, 22]. Cofilin regulates actin cytoskeletal reorganization by depolymerizing and severing actin filaments [21]. Due to the activity of cofilin is negatively regulated by phosphorylation at Ser3 and the phosphorylation site of cofilin localizes in the actin-binding domain, so the cytoskeleton dynamics was affected by the phosphorylation status of cofilin. Since the phosphorylation of LIMK2 and cofilin was affected by BMPR2 expression as discussed above, we hypothesized that there were some morphological changes when the alteration of BMPR2 levels. As shown in Figure 6A, prominent lamellipodial protrusions were found in the submembranous area of 143B-NC cells, but the opposite phenomenon was detected in 143B-shBMPR2 cells, which showed remodeled cytoskeleton and well distributed F-actin in cells. Similarly, in contrast with U2OS-control cells, the lamellipodial protrusions and stained F-actin filaments accumulated in the leading edge of BMPR2-overexpressed cells (Figure 6A). This suggests that the altered expression of BMPR2 could significantly affect cytoskeletal rearrangement in osteosarcoma cells.

Then, we evaluated whether the activation of LIMK2 was dependent on Smad molecules. As a BMP signal pathway inhibitor, LDN193189 (5 nmol/L) can block the BMP/Smad-dependent pathway. Decreased p-Smad1/5/8 level was detected in both shBMPR2 and LDN193189 groups compared with the control group (Figure 6B, *p*=0.011 and *p*<0.001, respectively). The phosphorylation of LIMK2 in shBMPR2 cells was less than the control group (Figure 6B, *p*=0.024); however, there was no change in the LDN193189 group (Figure 6B, *p*=0.082). In addition, 143B cells were transfected with siRNA targeting smad1 or smad5 prior to treatment with siBMPR2. As shown in Figure 6C, the suppressive effects of BMPR2 on p-LIMK2 expression levels were not affected by smad1 or smad5 knockdown in 143B cells. These results demonstrate that LIMK2 was activated through the BMP/Smad-independent pathway.

We performed an immunoprecipitation assay and found that BMPR2 could interact with LIMK2 directly in U2OS cells (Figure 6E). In addition, we detected the active pattern of RhoA through a GTPase activation assay and it was revealed that the more increased RhoA activation could be observed in BMPR2-overexpressed U2OS cells compared with control group (Figure 6D, *p*=0.032). Taken together, BMPR2 can activate LIMK2 through the small GTPase RhoA, and BMPR2 can also interact with LIMK2 in concert with changes in the downstream pathway.

### Silencing of BMPR2 suppresses osteosarcoma metastasis *in vivo*

**Figure 7:**
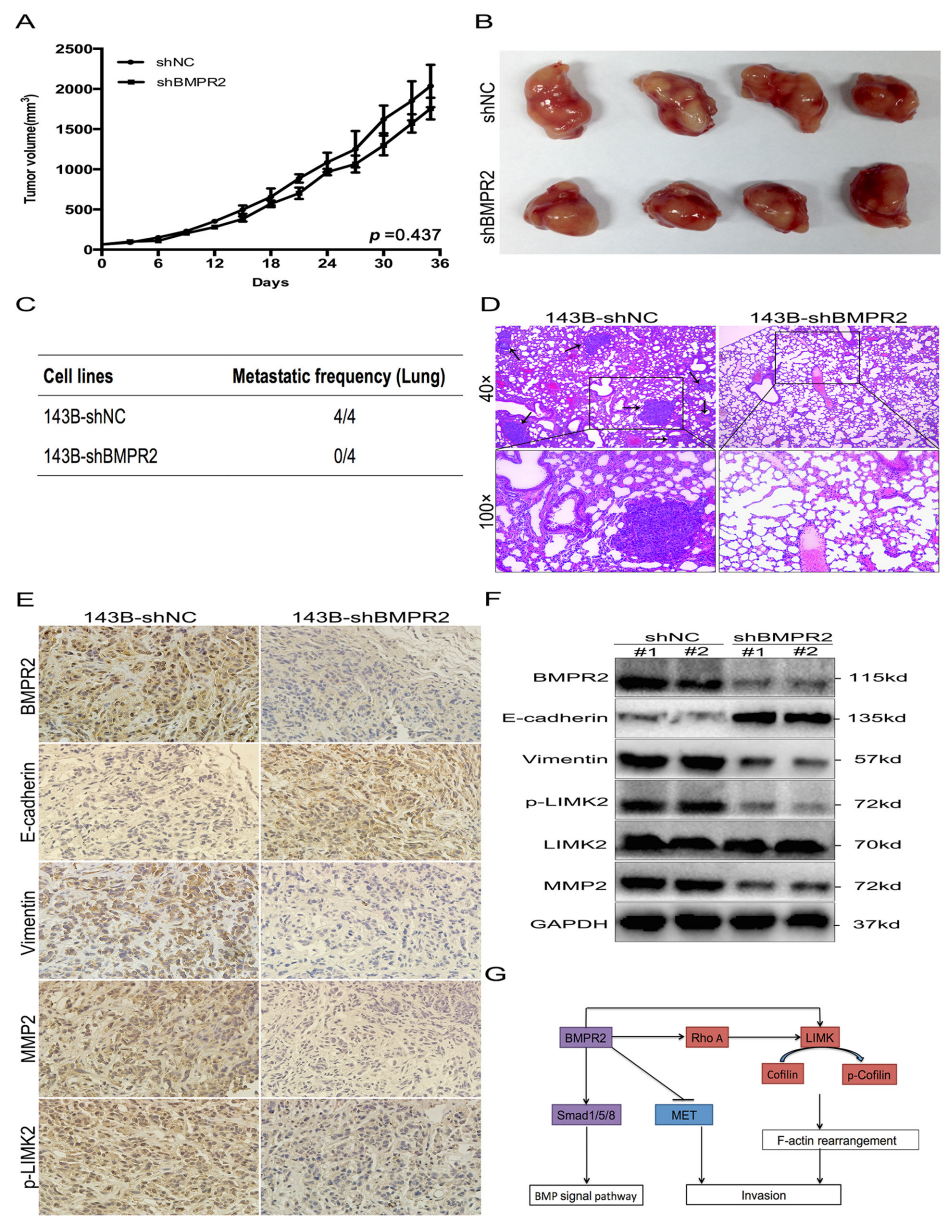
BMPR2-depletion suppressed osteosarcoma metastasis *in vivo* **(A)** The tumor growth curve of 143B-shBMPR2 and 143B-shNC groups. No significant difference was observed between the two groups. **(B)** Representative images of primary tumors. **(C)** The metastatic frequency of 143B-shNC cells and 143B-shBMPR2 cells. **(D)** H&E-staining of the lungs of the indicated 143B cells ± shBMPR2 group (magnification at ×40 and ×100). The arrows indicate the region of metastases. **(E)** IHC analysis of BMPR2, E-cadherin, vimentin, p-LIMK2 and MMP2. Images were photographed at ×400. **(F)** Changes in BMPR2, E-cadherin, vimentin, P-LIMK2 and MMP2 proteins were assayed by western blotting. **(G)** A model of BMPR2 in osteosarcoma invasion.

As BMPR2 silencing promoted MET and restrained *in vitro* migration and invasion of osteosarcoma cells, we further investigated whether BMPR2-depletion will affect *in vivo* growth and metastasis of tumor. Smaller primary tumor volume and lower growth rate were observed in shBMPR2 group than the shNC group, but the differences were not significant (Figure 7A and 7B, *p*=0.437). This showed that BMPR2 depletion has no effect on tumor formation *in vivo*.

Lung metastases were found in all four mice (4/4) in the 143B-shNC group, however, lung metastatic nodes were not found (0/4) in the 143B-shBMPR2 group (Figure 7C&Supplementary Figure 1). The representative images of the lungs after H&E staining were shown in Figure 7D. The numbers of micrometastases in the 143B-NC group were greater than the 143B-shBMPR2 group. These data suggest a function for BMPR2 as a pro-metastatic oncogene in osteosarcoma cells.

IHC and western blotting methods were used to detect invasion-related markers in primary tumors from both groups. In contrast with 143B-shBMPR2 primary tumors, the staining of BMPR2 in 143B-shNC tumors was more obviously positive (Figure 7E), which was possibly caused by significantly decreased BMPR2 expression in the BMPR2-silencing group (Figure 7F). Furthermore, IHC confirmed that tumors generated from BMPR2-silenced cells showed higher level of E-cadherin, but lower levels of vimentin, p-LIMK2 and MMP2 compared to tumors generated from control cells (Figure 7E).

The expressed changes in the corresponding factors according to western blotting were consistent with the IHC results, and the differences between the two groups were statistically significant (Figure 7F). In general, BMPR2 silenced 143B cells inhibited *in vivo* metastasis, but not primary tumor growth.

## DISCUSSION

Metastasis is the main factor affecting the prognosis of patients with osteosarcoma. It is a very complicated process that involves a variety of molecules and signal transduction pathways. Although the abnormal expression of BMPR2 has been detected in several cancers [12–17, 20], research on BMPR2 expression and the osteosarcoma metastatic mechanism is sparse. In this study, BMPR2 expression was found markedly elevated in osteosarcoma and this expression correlated with reduced overall and metastasis-free survival. Moreover, BMPR2-depletion decreased osteosarcoma cell invasion and metastasis *in vitro* and *in vivo* by the inactivation of the RhoA/ROCK/LIMK2 pathway (Figure 7G). Our results highlighted BMPR2 as an invasion and pro-metastasis indicator in osteosarcoma.

As the signal initiator, BMPR2 played a dominant role in BMP signaling pathway. Recent studies found a tendency towards lower BMPR2 level in metastatic prostate cancer than that in localized prostate cancer [23]. However, from analysis of BMPR2 mRNA levels and the clinical data, BMPR2 overexpression was correlated with metastases in osteosarcoma [20]. Thus, BMPR2 has a dual role in different tumors. In the current study, we confirmed that there is a significant correlation between BMPR2 overexpression and lung metastasis by immunohistochemistry method (Table 1, *p*=0.007). Independent of the effects on proliferation, the migration and invasive capacity was diminished in 143B cells accompanied with BMPR2 depletion. When BMPR2 was overexpressed in U2OS cells, the opposite data were obtained. Given that invasive capacity in BMPR2 has been found at both the tissue and cell levels, study of the role and mechanism of BMPR2 in osteosarcoma metastasis has important clinical value. In this research, we focused on the Smad-independent signaling pathway in osteosarcoma metastasis.

The epithelial-mesenchymal transition (EMT) was demonstrated in the metastatic process of several tumors [24–28]. As a complement, the mesenchymal-epithelial transition (MET) was shown to also be involved in complicated tumor metastasis [29–32]. Recently, many studies suggest that MET was also existed in mesenchymal tumors [33]. The exhibition of epithelial differentiation was reported in some soft tissue sarcomas, and the epithelial markers could be detected in several bone sarcomas. For example, the epithelial markers such as cadherin-11 and autocrine motility factor/ phosphoglucose isomerase (AMF/PGI) were expressed in osteosarcoma [33]. And a MET-like phenomenon in chondrosarcoma was observed through down-regulation of SNAI1 [34]. In our study, after BMPR2 exhaustion in 143B cells, their mesenchymal-like phenotype changed to an epithelial-like phenotype. Furthermore, BMPR2-depleted cells also showed increased E-cadherin and decreased N-cadherin and vimentin, both *in vitro* and *in vivo*. Similarly, some transcription factors such as Twist1 and ZEB1 were down-regulated, which was accompanied with BMPR2 inhibition. We confirmed this in U2OS cells. In addition, MMPs were regarded as important molecules assisting the invasion and metastasis of osteosarcoma cells. Wang et al [35] reported that EFEMP1-mediated osteosarcoma cells migration and invasion were linked to MMP-2. Connective tissue growth factor (CTGF) could promote osteosarcoma cells metastasis through the elevation of MMP-2 and MMP-3 [36]. Nobiletin inhibits human osteosarcoma cells motility, migration and invasion by down-regulating MMP-2 and MMP-9 expressions via ERK and JNK pathways [37]. Here, we found that the expression of MMP-2 was diminished after the BMPR2-depleted in 143B cells, so we suggest that MMP2 were involved in the BMPR2-induced MET in osteosarcoma cells. Although we provide a link between BMPR2 and MET in osteosarcoma, the mechanism of whether BMPR2 regulates MET directly or indirectly needs further study.

Protein phosphorylation has been demonstrated to be critical for tumor cell proliferation, cell survival and signal transduction [38–41]. As an important post-translational modification, phosphorylation can regulate protein activity [42]. Our quantitative phosphotyrosine proteomic analysis revealed that many pathways were involved in the Smad-independent pathway (Figure 5C). Among these, previous studies have shown that the actin-cytoskeletal pathway and focal adhesion pathway are significantly related with tumor metastasis [43–46].

Tumor cell invasion is a hallmark of metastasis, and that is a complex process involving cytoskeletal reorganization, lamellipodia formation, membrane ruffling and cell morphological changes [21]. The regulation of actin cytoskeleton plays an important role in cell migration, and LIMK and cofilin are crucial regulators. Actin reorganization is regulated by cofilin, which can be inactivated upon serine 3 phosphorylation by LIMK. The phosphorylation site of cofilin localizes in the actin-binding domain and inhibits its binding to actin filaments, completely blocking cofilin ability to promote filament disassembly. Therefore, cofilin activity regulated by its phosphorylation status determines the cytoskeleton dynamics. Several researches suggest that LIMK promotes the invasion of tumor cells by regulating the phosphorylation of cofilin [17, 21, 22, 47]. In human breast cancer cells, the overexpression of LIMK leads to an increase in invasive capacity [22]. Ding et al [21] showed that nischarin siRNA could enhance cofilin phosphorylation and stimulate breast cancer cell invasion. In addition, enhancement of LIMK increased the formation of invadopodia in colorectal cancer cells [17]. As shown in Figure 5D, BMPR2 silencing inhibits the phosphorylation of LIMK and cofilin. Rhodamine phalloidin staining showed that F-actin was mainly distributed 143B-shBMPR2 cell cytoplasm, but that F-actin and lamellipodial protrusions were accumulated at the edges of 143B-control cells. Similarly, the phosphorylation of LIMK2 and cofilin was enhanced by BMPR2 overexpression in U2OS cells, and aberrant F-actin assembled under the submembrane. Combined with the anti-invasion capacity of BMPR2 silencing, we speculate that BMPR2 inhibition may lower cofilin phosphorylation and elevate cofilin activation, resulting in reduces lamellipodia extension and cell invasion. Moreover, we found that activation of RhoA was involved in the LIMK/cofilin pathway in osteosarcoma cells. The immunoprecipitation assay revealed that BMPR2 interacted with LIMK2 directly. However, there is an inconsistent link between LIMK status and BMPR2 in previous studies. Foletta VC et al [48] reported that BMPR2 inhibited LIMK's ability to phosphorylate cofilin in the pathology of primary pulmonary hypertension. Voorneveld et al [17] showed that LIMK activation was activated by BMPR2 in colorectal cancer cells. For the first time, we showed that BMPR2 was a positive regulator of LIMK2 in osteosarcoma cells. We also confirmed the decreased expression of p-LIMK in BMPR2-depleted tumors from nude mice compared to controls.

In summary, BMPR2 functions as a prometastatic oncogene. The silencing of BMPR2 inhibits invasion and metastasis by the RhoA/ROCK/LIMK pathway, and that also indicates a potential therapeutic target for osteosarcoma.

## MATERIALS AND METHODS

### Bioinformatics analysis of BMPR2 expression in osteosarcoma

To determine BMPR2 gene expression in osteosarcoma, we analyzed datasets generated from published studies in the R2.12.0 platform (http://r2.amc.nl) (Academic Medical Center, Netherlands). 39 samples in the microarray data (GSE33383) were removed from the analysis because of lacking survival data. The remaining 88 samples were included in the research. A log-rank test was applied in order to investigate the Kaplan–Meier survival (Harrington and Fleming, 1982).

### Osteosarcoma patients’ information gathering and tissue samples collection

Twelve resected osteosarcoma and twelve normal bone tissue samples were involved in this research. Bone tissues were collected following the protocols provided by Peking University People's Hospital, and was approved by their independent ethics committee (Beijing, China). Written informed consent was obtained from all subjects. In addition, we examined paraffin-embedded tissue specimens with osteosarcoma at the Department of Pathology in Peking University People's Hospital. Specimens without prior treatment were included in this research (n=67). Follow-up information was gathered through outpatient visits and telephone calls, and was ended by June 15, 2016. Follow-up lasts from 6 to 105 months with a median of 62.5 months.

### Immunohistochemistry

Immunohistochemistry (IHC) staining was conducted as described previously [16]. According to the staining positive percentage of osteosarcoma cells, the sections were graded by four levels: 0: 0% positive; 1: < 5% positive; 2: 5-50% positive; and 3: > 50% positive. Staining intensity was also graded by four levels: 0: none; 1: mild staining; 2: moderate staining; and 3: intense staining. Total score = percentage score × staining intensity. Based on the median staining score, sections were divided into a high and low expression group.

### Cell culture

HOS, KHOS, 143B, MNNG, U2OS, and SAOS2 cells were purchased from the American Type Culture Collection (ATCC). Cell culture medium contained 10% fetal bovine serum (Gibco) and 1% penicillin/streptomycin (Invitrogen). 143B, MNNG and SAOS2 cells were cultured in DMEM/ high glucose medium (Invitrogen). HOS, KHOS and U2OS cells were cultured with RPMI 1640 medium (Invitrogen). Cells lines were maintained at 37 °C with 5% CO_2_.

### Recombinant lentivirus and adenoviral expression vectors and cell transfections

A lentivirus construct targeting BMPR2 and a non-targeting lentivirus construct as control were obtained from the Hanbio Technology Company (Shanghai, China). Two pairs of shBMPR2 sequences were designed: pair 1: sense strand 5′-GCCTATGGAGTGAAATTATTT-3′ and antisense strand 5′-AAATAATTTCACTCCATAGGC-3′; pair 2: sense strand 5′-CCTAACTGTATACCAGAATTA-3′ and antisense strand 5′-TAATTCTGGTATACAGTTAGG-3′. shNC sequences were presented as follows: sense strand: 5′-TTCTCCGAACGTGTCACGTAA-3′ and antisense strand: 5′-TTACGTGACACGTTCGGAGAA-3′. For transient overexpression of BMPR2 (NM_001204), an adenoviral vector of full human genomic BMPR2 DNA fused with enhanced green fluorescent protein (EGFP) gene driven by cytomegalovirus promoters (pAd-CMV-BMPR2) was constructed. Adenoviral vector containing EGFP gene only was constructed as negative control. Recombinant viruses were collected and purified, and titer was determined following the manufacturer's instructions.

For lentiviral transfection, 143B cells (2 × 10^5^) were seeded onto six-well culture plates. Sixteen hours later, cells were transduced with LV-shBMPR2 or LV-shNC. 48 hr after viral transduction, green fluorescence (from ZsGreen) was captured by fluorescence microscope (Olympus IX81, Tokyo, Japan). Stable transfected cell lines were obtained upon 5 μg/ml puromycin (Sigma) selection and were maintained in medium containing 2 μg/ml puromycin for 14 days. In addition, to investigate the effect of BMPR2 overexpression in U2OS cells, BMPR2 adenovirus or empty vectors were administered by transient transfection. Adenoviral infection efficiency was evaluated at 48 hr post infection according to gathered GFP signal.

### Gene expression analysis by real-time PCR, western blotting and GTPase assay

Total RNA was extracted by using Trizol (Invitrogen) as instructed by manufacturer. Complementary DNA (cDNA) was synthesized using 500 ng total RNA, OligdT primers and SuperScriptIII reverse transcriptase (Invitrogen). Real-time PCR system was prepared using SYBR Green I (Takara, Dalian, China) and primers listed in Supplementary Table 1, and was run on Bio-Rad CFX96 (Applied Biosystems, California, USA). Relative transcript expression values were extrapolated by normalizing to GAPDH as internal control.

Western blotting was performed as previously described in our publication [49]. Anti-human BMPR2 (ab78422), total-Smad1/5 (ab75273), p-LIMK2 (ab38499), LIMK2 (ab97766), MMP2 (ab92536), p-MEK (ab96379), MEK (ab32091), p-FAK (ab39967), FAK (ab61113) and Twist (ab135180) antibodies as well as the RhoA activation assay (ab211164) were purchased from Abcam (Cambridge, UK). The inhibitor of LDN193189 was purchased from Selleck (Houston, Texas, USA). Antibodies against E-cadherin (no. 3195), N-cadherin (no.13116), Vimentin (no. 5741), ZEB1 (no. 3396), p-cofilin (no.3313), cofilin (no.5175), p-GSK3β (no.9322), GSK3β (no.12456), p-Smad1/5 (no. 9516), smad1 (no.6944) and smad5 (no.12534) were purchased from Cell Signaling Technology (Beverly, MA, USA). Anti-GAPDH and HRP-conjugated secondary antibodies were purchased from ZSGB-BIO (Beijing, China). The protocol for the GTPase assay was followed as per the kit instructions.

### *In vitro* growth curves

To observe cell growth, 1×10^4^ cells were seeded onto a 12 well plate. From day 2 to day 7, cells counting were recorded after counted with hematocytometer at the same time point every day.

### Cell viability assay

Cells were seeded in a 96-well plate at a concentration of 5000 cells per well before experiment. After 48h of BMPR2 transfection, cell viability was assessed by MTS (3-(4,5-dimethylthiazol-2-yl)-5-(3-carboxymethoxyphenyl)-2-(4-sulfophenyl)-2H-tetrazolium) assay as described previously [49].

### Wound healing assay

In order to evaluate 143B and U2OS cell mobility, confluent osteosarcoma cells in a 6-well plate were scratched carefully using 200 μl sterile pipette tips, and cell debris was discarded. Images were taken at 0 and 24 h and analyzed using Image J software (Rawak Software, Inc. Germany).

### Transwell assay

1×10^5^ cells were seeded into the non-coated upper chamber for migration capacity and matrigel coated transwell inserts with 8.0 μm filters (Corning) for invasiveness. After culturing for 24 hr, cells were fixed by methanol and stained with 0.5% crystal violet staining solution. Migrated cell population was evaluated by Image J software (Rawak Software, Inc. Germany).

### Sample preparation, iTRAQ labeling and LC-MS/MS analysis

The buffer containing 4% SDS, 100 mM DTT, and 150 mM Tris-HCl pH8.0 was prepared for protein extraction and digestion. The total proteins were exacted from the cells. Desalted peptides were labeled with isobaric tags for relative and absolute quantitation (iTRAQ) reagents: 143B-shNC with reagent 114, 143B-shBMPR2 with reagent 115, U2OS-pcDNA with reagent 116, and U2OS-BMPR2 with reagent 117. Phosphopeptide enrichment was carried out using a TiO_2_ column. In addition, the non-phosphopeptides that were not retained were removed. The dried phosphopeptides were analyzed directly on Thermo Q Exactive MS (Thermo Scientific, Massachusetts, USA). Two independent biological replicates were performed. The data for the phosphopeptides in two biological replicates were combined, and the average of the same phosphopeptides was calculated. Ratios of 115:114 and 117:116 of phosphopeptides were calculated, and data normalization was log_2_-transformed. According to previous research [42, 50], the phosphorylation changes were considered significant if the increased or decreased fold change >1.5 and the *p*-value <0.05. The identification of labeled phosphopeptides was analyzed using Mascot 2.2 (Matrix Science, London, UK) and Proteome Discoverer 1.4 (Thermo Scientific, Massachusetts, USA).

### Immunofluorescence assay

For immunofluorescence, cells (2 × 10^5^ cells per well) were adhered to coverslips in six-well plates. Cells were then fixed in 4% paraformaldehyde for 20 min and incubated in PBS with 0.1% Triton X-100 for 5 min. Coverslips were then moved to a piece of parafilm in a humid chamber to which 200 μl of 100 nM rhodamine phalloidin was added (Cytoskeleton Inc. Denver, USA). They were then incubated at room temperature shielded from light for 45 min. Nuclei were stained with PBS with 2 μg/ml Hoechst 33342 for 4 min. Staining was photographed by Leica TCS SP5 laser scanning spectral confocal microscope (Leica, Mannheim, Germany).

### Immunoprecipitation

After 48 h of the adenoviral transfection, U2OS cells were lysed in pre-cooled lysis buffer end-over-end for 30 min at 4°C. The supernatant was extracted via centrifugation for 15 min at 13000 rpm (4°C). Flag beads (A2220, Sigma) (30 μl) were added and rotated for 3 h at 4°C, and washed four times by pre-cooled lysis buffer. Afterwards, the pellet was incubated with 2×loading buffer (40 μl) and heated to 95°C for 5 min. Then, the immunoprecipitated protein was visualized by western blotting.

### Tumor xenografts

BALB/c nude mice at 4 to 6 weeks of age were purchased from the Experimental Animal Center of Vitalriver Company (Beijing, China) and raised under SPF conditions. Mice were orthotopically injected with 5 × 10^6^ 143B- shBMPR2 and shNC cells into the para-osseous proximal tibia. Since day 7 after injection, tumor length and width were measured using Vernier caliper every 3 days. Tumor volume was calculated as (length × width^2^)/2. Mice were sacrificed at the fifth week and tumor-bearing limbs were collected, weighed, and photographed [51, 52]. Tumor specimens were preserved at -80°C for further study. To visualize the number of lung metastatic nodules, 10% formalin fixed and paraffin embedded lung samples were sectioned at sequentially at 3-μm-thick, and then the sections were stained with H&E to identify the metastases by light microscopy. Animal involved procedures were performed following Peking University People's Hospital approved protocols.

### Statistical analysis

Statistical analysis was conducted by SPSS17.0 software package (SPSS Inc., Chicago, IL, USA). Analysis of survival and difference between the two groups was performed using Kaplan-Meier method and log-rank test. Correlation between BMPR2 expression and clinical pathology features was assessed by Chi-squared test. Multivariate survival analysis was performed using the Cox proportional hazard model. Multiple comparisons were evaluated using one-way ANOVA, and comparison among the groups was performed with SNK test. Two groups comparisons were performed using two-tailed Student's *t-*test. Data were represented as the mean ± S.D. Differences with *P* <0.05 was considered as statistically significant.

## SUPPLEMENTARY MATERIALS FIGURES AND TABLES




