# Supplementary material for: BMPR2 promotes invasion and metastasis via the RhoA-ROCK-LIMK2 pathway in human osteosarcoma cells
Source: Oncotarget. 2017 Apr 24;8(35):58625–41. doi: 10.18632/oncotarget.17382 (PMC5601680; doi:10.18632/oncotarget.17382)
Supplement: Supplementary file 1 [file oncotarget-08-58625-s001.pdf]

BMPR2 promotes invasion and metastasis via the RhoA-ROCK-LIMK2 pathway in human osteosarcoma cells

SUPPLEMENTARY FIGURE AND TABLES

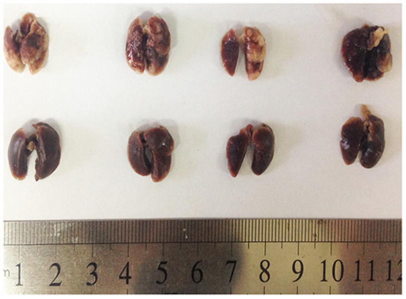

Supplementary Figure 1: Photographs of lungs of each group.

Supplementary Table 1: Primers used for quantitative real-time PCR

Gene	Primer direction	Sequence
BMPR2	Forward	5′-CACTCAGTCCACCTCATTCATTT-3′
	Reverse	5′-TTGTTTACGGTCTCCTGTCAAC-3′
E-cadherin	Forward	5′-GCTGGAGATTAATCCGGACA-3′
	Reverse	5′-ACCTGAGGCTTTGGATTCCT-3′
N-cadherin	Forward	5′-CCACCTTAAAATCTGCAGGC-3′
	Reverse	5′-GTGCATGAAGGACAGCCTCT-3′
Vimentin	Forward	5′-CTGGATTTCCTCTTCGTGGA-3′
	Reverse	5′-CGAAAACACCCTGCAATCTT-3′
Twist1	Forward	5′-TCCATTTTCTCCTTCTCTGGAA -3′
	Reverse	5′-CCTTCTCGGTCTGGAGGAT -3′
MMP2	Forward	5′-GGAAAGCCAGGATCCATTTT -3′
	Reverse	5′-ATGCCGCCTTTAACTGGAG-3′
ZEB 1	Forward	5′-AAGAATTCACAGTGGAGAGAAGCCA-3′
	Reverse	5′-CGTTTCTTGCAGTTTGGGCATT-3′
GAPDH	Forward	5′-GCACCGTCAAGGCTGAGAAC-3′
	Reverse	5′-ATGGTGGTGAAGACGCCAGT-3′

The mRNA levels of MET markers and MMP2 were investigated by using the following primers. GAPDH severs as control.

Supplementary Table 2: The combined phosphopeptides that were regulated by BMPR2, as quantified by iTRAQ analysis.

See Supplementary File 1
