# Supplementary material for: BMPR2 promotes invasion and metastasis via the RhoA-ROCK-LIMK2 pathway in human osteosarcoma cells
Source: Oncotarget. 2017 Apr 24;8(35):58625–41. doi: 10.18632/oncotarget.17382 (PMC5601680; doi:10.18632/oncotarget.17382)
Supplement: Supplementary file 2 [file oncotarget-08-58625-s002.docx]

**Supplementary Table 2****:** The combined phosphopeptides that were regulated by BMPR2, as quantified by iTRAQ analysis.

| Accession number | Protein name | Sequence^1^ | Phosphosite(s) | 115:114^2^ | 117:116^3^ |
| --- | --- | --- | --- | --- | --- |
| Q05397 | PTK2 | sVsEtDD**y**AEIIDEE | Y397 | 0.384 | 1.657 |
| P67775 | PPP2CA | VTRRtPD**y**FL | Y307 | 0.442 | 1.904 |
| P67775 | PPP2CA | EPHVTRR**t**PDyFL | T304 | 0.316 | 1.722 |
| P49841 | GSK3β | SGRPRtt**s**FAEsCKP | S9 | 1.831 | 0.467 |
| P36897 | TGFβR1 | VPNEEDP**s**LDRPFIs | S165 | 0.452 | 1.914 |
| Q9NYB9 | ABI2 | PPVVPND**y**VPsPtRN | Y213 | 2.602 | 0.402 |
| P61586 | RhoA | ARRGKKK**s**GCLVL | S188 | 0.405 | 3.601 |
| Q02750 | MAP2K1 | RtPGRPL**s**SyGMDSR | S298 | 0.611 | 1.636 |
| Q06330 | RBPJ | EPPAHAP**s**PGKFGER | S18 | 2.359 | 0.606 |
| O15111 | CHUK | AKDVDQG**s**LCTsFVG | S176 | 1.558 | 0.593 |
| P46937 | YAP1 | PQHVRAH**s**sPAsLQL | S127 | 0.475 | 2.019 |
| Q9UJ41 | RABGEF1 | PSINRQt**s**IEtDRVS | S310 | 0.424 | 1.712 |
| Q01860 | POU5F1 | LASDFAF**s**PPPGGGG | S12 | 0.498 | 1.583 |
| P53671 | LIMK2 | NDRKKRY**t**VVGNPY | T505 | 0.396 | 3.142 |
| P00533 | EGFR | DsFLQRy**s**sDPtGAL | S1070 | 1.764 | 0.446 |
| P00533 | EGFR | sFLQRys**s**DPtGALt | S1071 | 2.035 | 0.414 |
| P58107 | EPPK1 | RGLRRQV**s**AsELHTs | S2716 | 0.627 | 1.604 |
| Q9Y6W5 | WASF2 | PKRssVV**s**PsHPPPA | S296 | 1.548 | 0.447 |
| P45983 | MAPK8 | AGtsFMM**t**PyVVtRY | T183 | 1.524 | 0.575 |
| Q86WA8 | LONP2 | MPQSMPE**y**ALTRNY | Y307 | 0.647 | 0.510 |
| P31749 | AKT1 | KDGAtMK**t**FCGtPEy | T308 | 0.426 | 1.534 |
| P63000 | RAC1 | yDRLRPL**s**YPQTDVF | S71 | 1.592 | 0.422 |
| P04732 | MTIE | SCKKSCC**s**CCPVGCA | S35 | 1.798 | 0.641 |
| P13645 | KRT10 | LNDRLAs**y**LDKVRAL | Y160 | 1.625 | 0.527 |
| P51812 | RPS6KA3 | DHEKKAy**s**FCGtVEy | S227 | 0.467 | 1.521 |
| Q8WUW1 | BRK1 | ALERRIE**y**IEARVTk | Y63 | 3.704 | 0.436 |
| P69905 | HBA1 | VGAHAGE**y**GAEALE | Y25 | 1.584 | 0.586 |
| Q8WUY8 | NAT14 | GLRARWG**S**LPPPGG | S87 | 0.631 | 1.505 |
| Q9GZR2 | REXO4 | ASKRAPs**s**PVAKPGP | S15 | 2.222 | 0.576 |
| P25391 | LAMA1 | HNKAKDC**y**YDESVA | Y337 | 0.533 | 1.634 |
| Q16539 | MAPK14 | RHtDDEM**t**GyVAtRW | T180 | 1.545 | 0.622 |
| Q03135 | CAV1 | VDsEGHL**y**tVPIREQ | Y14 | 0.418 | 1.631 |
| P23528 | CFL1 | MA**s**GVAVsDG | S3 | 0.251 | 1.989 |
| Q96D46 | NMD3 | AIPVEsD**t**DDEGAPR | T470 | 1.511 | 0.538 |
| P05230 | FGF1 | VGLKKNG**s**CKRGPR | S131 | 0.332 | 2.349 |
| Q05209 | PTPN12 | FMRLRRL**s**tKYRTEK | S39 | 1.612 | 0.534 |
| Q03692 | COL10A1 | KPGQQGP**T**GAPGPR | T164 | 0.492 | 1.528 |
| Q71DI3 | HIST2H3A | ATKAARK**s**APATGG | S29 | 0.572 | 0.611 |
| P11362 | FGFR1 | ALTSNQE**y**LDLSMPL | Y766 | 1.702 | 0.404 |
| Q99538 | LGMN | MYRKMVF**y**IEACES | Y185 | 0.558 | 1.522 |
| Q13043 | STK4 | GDyEFLK**s**WtVEDLQ | S438 | 0.444 | 2.162 |
| P0DMS8 | ADORA3 | DTSIEKN**S**E | S317 | 1.627 | 0.603 |
| Q5JRX3 | PITRM1 | NGYTREA**y**FSVGLQ | Y388 | 0.586 | 0.632 |
| P02795 | MT2A | MDPNC**s**CAAGDsC | S6 | 0.574 | 1.581 |
| P78395 | PRAME | ERRRLWG**s**IQSRYIs | S9 | 0.617 | 1.642 |

^1^ The third column represents the primary sequence of the phosphopeptide identified. And the aminoacid modified by phosphorylation was marked in bold.

^2^ The number of 114 and 115 represent the cells of 143B-NC and 143B-shBMPR2, respectively.

^3^ The number of 116 and 117 represent the cells of U2OS-pcDNA and U2OS-BMPR2, respectively.
